# Atypical Origin of the Pectoral Nerves: Morphological Insights for Orthopedic, Reconstructive, and Trauma Surgeons

**DOI:** 10.7759/cureus.109489

**Published:** 2026-05-23

**Authors:** José A Rosario Gonzalez, Gabriella D Torres Irizarry, Shay M Blanco Olivares, Natalia Valentin Carro

**Affiliations:** 1 School of Medicine, University of Puerto Rico, Medical Sciences Campus, San Juan, PRI; 2 Anatomy and Neurobiology, University of Puerto Rico, Medical Sciences Campus, San Juan, PRI

**Keywords:** ansa pectoralis, brachial plexus anatomical variations, clavicle fixation, lateral pectoral nerve, medial pectoral nerve, nerve anastomosis, orthopedic surgery, pectoralis major muscle, pectoralis major tendon repair, shoulder arthroplasty

## Abstract

Anatomical variations in the brachial plexus can significantly affect upper-extremity surgical procedures. This report highlights a rare case where both pectoralis muscles receive accessory innervation. The pectoralis major exhibited three additional branches stemming directly from the anterior divisions of the superior and middle trunks, effectively bypassing the lateral cord. The pectoralis minor likewise demonstrated variant innervation, receiving two additional branches. One was an unexpected confluence in which a branch destined for the pectoralis major coursed through and innervated the pectoralis minor, despite the pectoralis minor typically receiving its own independent branch. The other branch originated from the anterior division of the middle trunk. Furthermore, a variant ansa pectoralis was identified, originating from the anterior division of the middle trunk and medial cord, and giving rise to the medial and lateral pectoral nerves. This unique configuration may present clinical implications for surgeons, particularly during procedures involving the proximal upper limb and supraclavicular pectoral region. Identifying these variations can help minimize the risk of iatrogenic nerve injury and enhance surgical planning.

## Introduction

The brachial plexus is a complex network of nerves essential for motor and sensory functions in the upper limbs [1,2]. The plexus originates from the ventral rami of spinal nerves C5 to T1, forming roots that extend into the developing arm bud, navigating around structures such as blood vessels, cartilage, and bones [1-3]. It develops during embryogenesis through a detailed process involving nerve migration and fusion [4]. As the nerves migrate, they merge into the plexus, initially forming three trunks, superior, middle, and inferior, that then split into six anterior and posterior divisions [5,6]. These divisions reorganize into three cords, medial, lateral, and posterior, named according to their position relative to the axillary artery [5,6]. Ultimately, the plexus develops into five terminal branches, musculocutaneous, axillary, radial, median, and ulnar nerves, that provide the motor and sensory functions of the upper limb.

During its development, the plexus can exhibit anatomical variations due to abnormal neural crest migration, disruptions in molecular signaling, genetic factors regulating axonal guidance and limb patterning, aberrant positioning of somite-derived myogenic precursor cells, or spatial constraints imposed by adjacent developing structures [7-11]. Common variations include communications between the musculocutaneous and median nerves, particularly where the musculocutaneous nerve receives branches from the median nerve, and differences in root contributions, such as from C4 or T2 nerve roots [1,12,13]. Harry et al. reported that variability in brachial plexus branching patterns occurs in approximately 16% of trunks, 4% of cords, and 25% of terminal branches, with terminal branches being the most variable [14-16]. Researchers note that variations of the pectoral nerves, which originate from the anterior divisions of the trunks, are less frequently described because both nerves typically arise from the lateral and medial cords [17]. However, cadaveric studies by Prakash and Saniya show that trunk-level nerve variations can occur, with the lateral pectoral nerve arising from the upper and middle trunk's anterior divisions in about 10% of cases and the medial pectoral nerve originating from the lower trunk's anterior division in up to 43.8% of cases [17]. These findings emphasize that variations, although not often highlighted, can have significant clinical implications.

## Case presentation

During a cadaveric dissection of the right axillary region of a formalin-fixed elderly male at the School of Medicine, University of Puerto Rico, an unusual variation of the brachial plexus was identified. The brachial plexus was exposed by sequentially dissecting the root of the neck and axilla, retracting both pectoralis muscles from their attachments, and tracing each component of the plexus using the clavicle and axillary vessels as landmarks. Special focus was given to the anterior divisions of the lateral and middle trunks after unexpected branches appeared early in the dissection (Table 1).

**Table 1:** Comparison between typical pectoral nerve anatomy and the variant anatomy observed in this case. LPN: lateral pectoral nerve; MPN: medial pectoral nerve

Nerve	Typical anatomy (origin)	Variation observed in this case
LPN	Side branch of the lateral cord of the brachial plexus	Arises from a variant trunk formed by contributions from the anterior division of the superior trunk and the anterior division of the middle trunk
MPN	Side branches from the medial cord of the brachial plexus	Forms a loop (expanded ansa pectoralis) between the anterior division of the middle trunk and the medial cord

**Figure 1:**
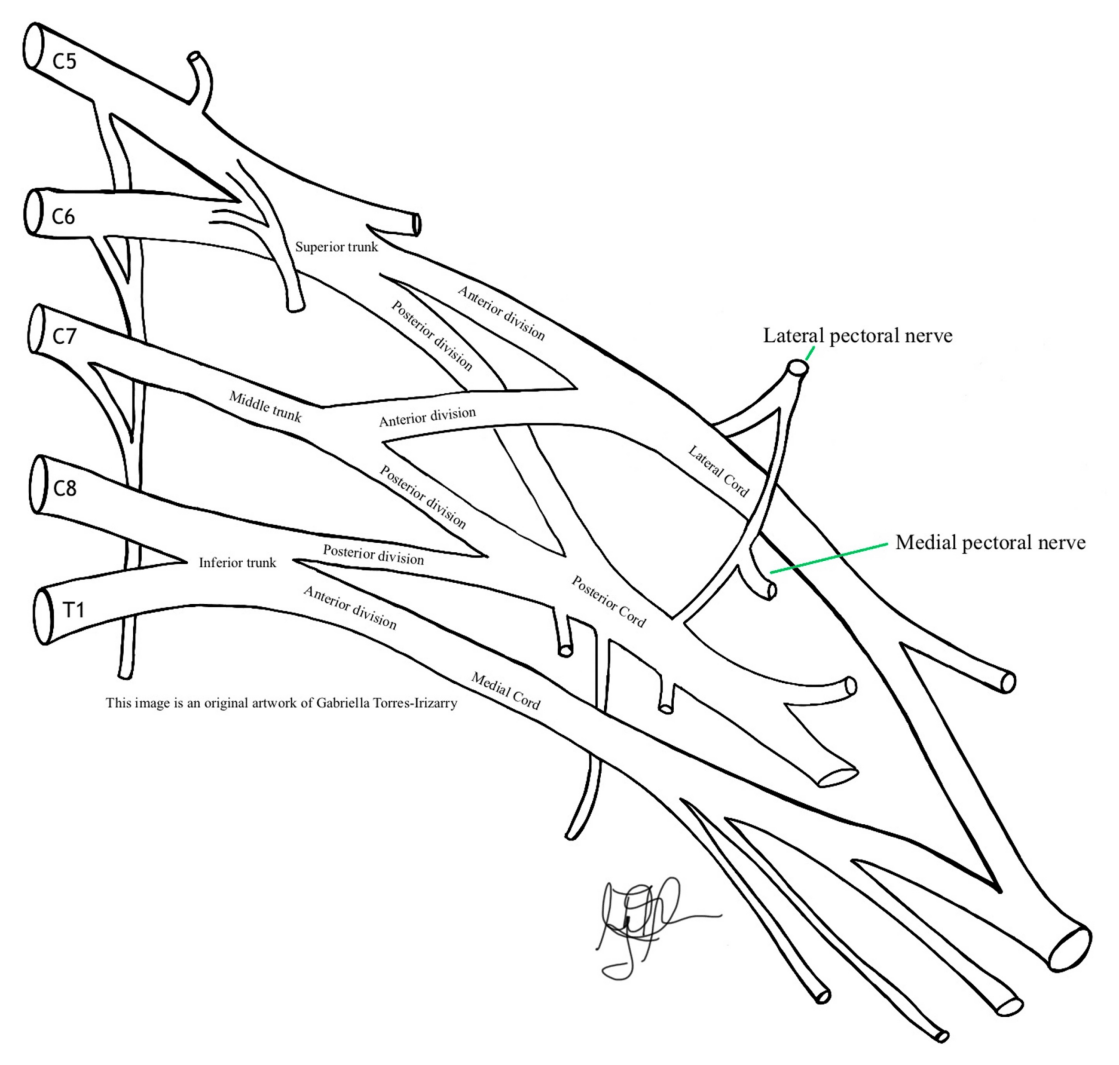
Schematic representation of the normal brachial plexus anatomy. This original schematic diagram illustrates normal brachial plexus anatomy (black). This schematic illustration is an original drawing created by the author (Gabriella Torres Irizarry) of this study using the Procreate software (Hobart, Australia: Savage Interactive Pty Ltd).

**Figure 2:**
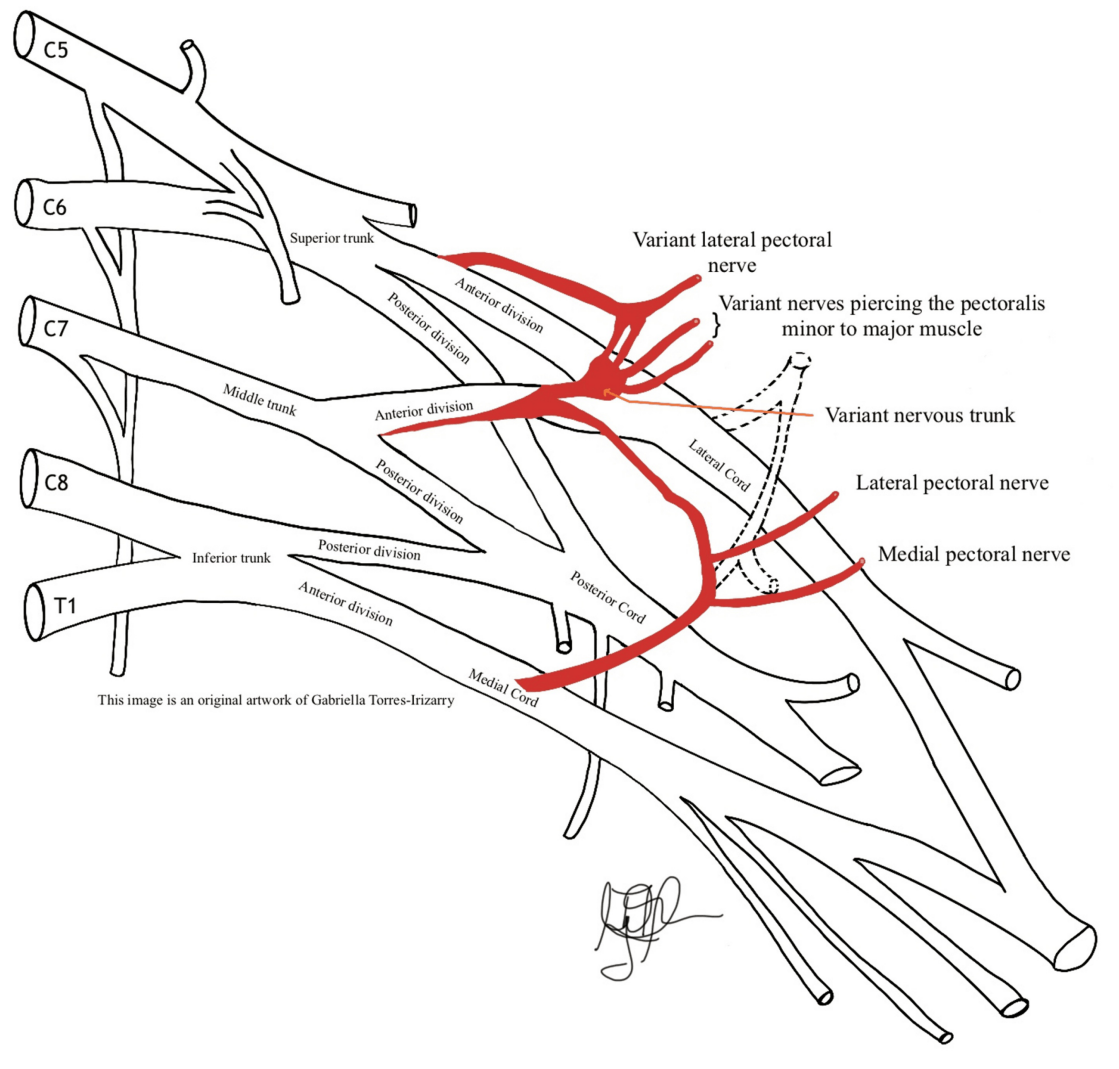
Schematic representation of the variant origins overlapping normal brachial plexus anatomy. This original schematic diagram illustrates normal brachial plexus anatomy (black) and the variant branching pattern identified in this case (red). The diagram highlights the relationship of the variant lateral pectoral nerve with the superior trunk and anterior divisions of the brachial plexus. The illustration was produced from direct anatomical observations during the cadaveric dissection performed at the University of Puerto Rico Medical Sciences Campus, San Juan, Puerto Rico. This schematic illustration is an original drawing created by the author (Gabriella Torres Irizarry) of this study using the Procreate software (Hobart, Australia: Savage Interactive Pty Ltd) based on the cadaveric dissection described in this report.

A detailed inspection revealed that a variant lateral pectoral nerve originated directly from the anterior division of the superior trunk rather than from the lateral cord. This unusual branch joined a robust variant of the neural trunk arising from the anterior division of the middle trunk, forming an uncommon confluence not described in typical anatomical literature (Figures 1, 2).

**Figure 3:**
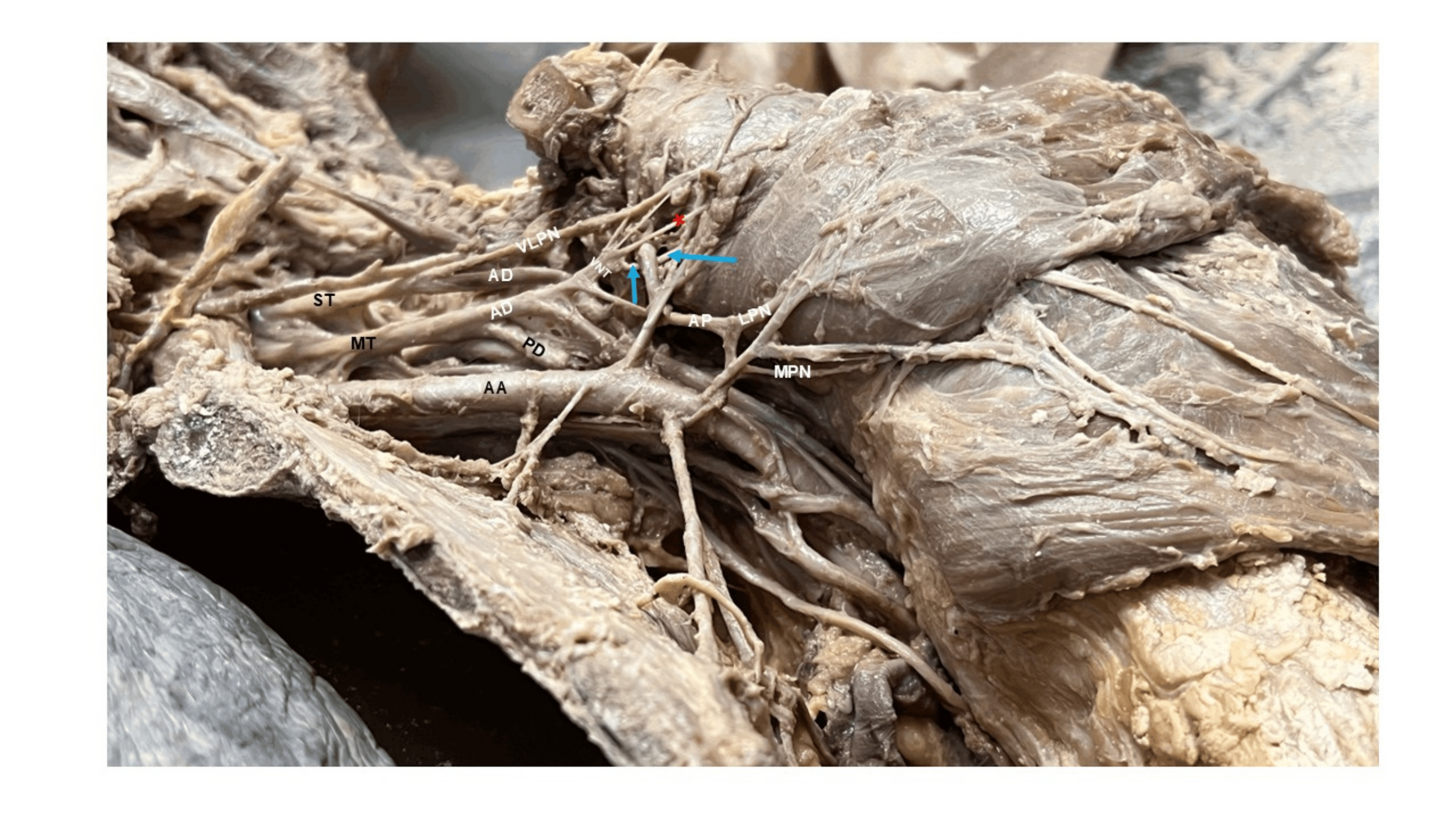
Original cadaveric dissection demonstrating the variant anomalous pectoral nerve branching pattern of the brachial plexus. This original cadaveric photograph, obtained during the dissection performed, demonstrates the relationship between the brachial plexus and the pectoral nerve branches identified in this specimen. The blue arrows indicate the variant nervous trunk arising proximal to the typical origin of the lateral pectoral nerve. The red asterisk marks the branch piercing the pectoralis minor muscle before continuing toward the pectoralis major, representing the variant innervation pattern observed in this case. VLPN: variant lateral pectoral nerve; ST: superior trunk; MT: middle trunk; VNT: variant nerve trunk; LPN: lateral pectoral nerve; AA: axillary artery; MPN: medial pectoral nerve; AD: anterior division; PD: posterior division; AP: ansa pectoralis; red asterisk: additional nerve to the pectoralis major; blue arrows: nerve that pierces the pectoralis minor to the pectoralis major

This variant neural trunk gave rise to the following five branches: two branches merged with the variant lateral pectoral nerve from the superior trunk to form a combined pectoral pathway to the pectoralis major; the third branch traveled directly to the pectoralis major muscle; the fourth branch pierced the pectoralis minor en route to the pectoralis major; and a final branch traveled medially to unite with the medial cord and form an extended ansa pectoralis that crossed anterior to the axillary artery and vein (Figures 2, 3).

**Figure 4:**
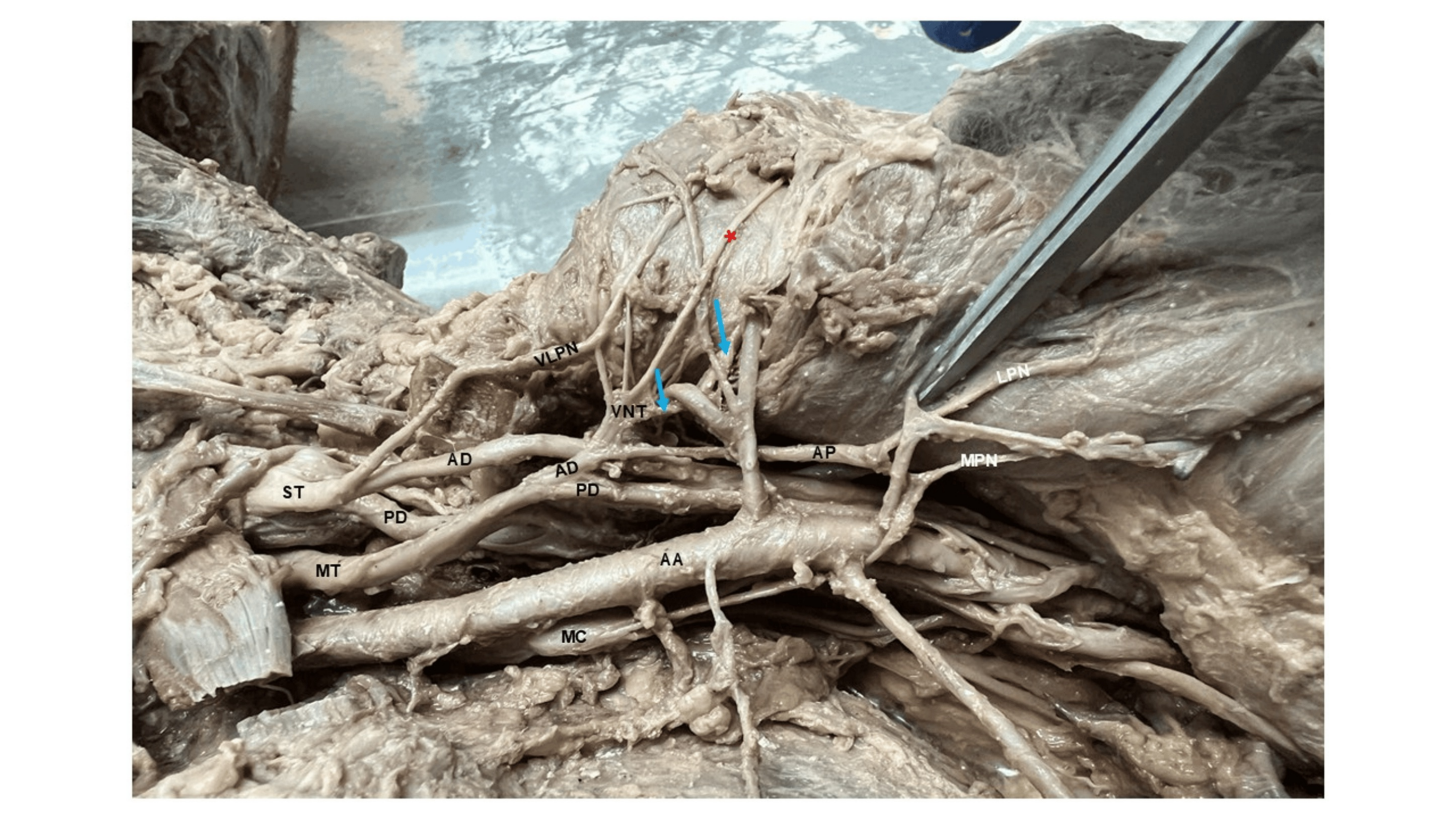
Cadaveric dissection demonstrating an extended ansa pectoralis arising from the anterior division of the middle trunk and medial cord. This original cadaveric photograph, obtained during the dissection performed, highlights the formation of an extended ansa pectoralis (AP) connecting branches derived from the anterior division of the middle trunk and the medial cord of the brachial plexus. The blue arrows indicate the variant nervous trunk contributing to this communication. The red asterisk marks the branch that pierces the pectoralis minor muscle before continuing toward the pectoralis major. This configuration demonstrates an expanded neural loop contributing to the pectoral nerve network. VLPN: variant lateral pectoral nerve; ST: superior trunk; MT: middle trunk; VNT: variant nerve trunk; LPN: lateral pectoral nerve; AA: axillary artery; MPN: medial pectoral nerve; AD: anterior division; PD: posterior division; MC: medial cord; AP: ansa pectoralis; red asterisk: additional nerve to the pectoralis major; blue arrows: nerve that pierces the pectoralis minor to the pectoralis major

Notably, the ansa pectoralis received no contribution from the lateral cord, deviating from the expected cord-level pattern. Instead, it arose solely from the anterior division of the middle trunk and the medial cord, giving rise to both the lateral and medial pectoral nerves (Figure 4). In this specimen, the lateral pectoral nerve pierced the pectoralis minor before terminating in the pectoralis major, while the medial pectoral nerve coursed along the lateral border of the pectoralis minor, pierced it, and also supplied the pectoralis major. As a result, all variant branches ultimately contributed to the innervation of the pectoralis major.

No other neurovascular anomalies were observed, and the pectoral muscles appeared normally developed and appropriately innervated. All findings were documented through direct visualization, high-resolution photography, and schematic illustration, and subsequently verified by faculty anatomists.

## Discussion

The brachial plexus is a complex network formed through a series of coordinated embryological processes, including neural crest migration, axonal growth, and fusion. This organization results in the ventral rami of C5-T1 forming roots, trunks, divisions, cords, and terminal branches that supply the upper limb [1-3,18]. Minor disruptions during development or in the positioning of nearby musculoskeletal structures can lead to variants in neural branching patterns [7-10]. Most variations involve communications between the musculocutaneous and median nerves or atypical root contributions [1,11,12]. However, fewer reports describe abnormal origins of the pectoral nerves from early trunk or division levels of the plexus [13,16,17]. While no specific syndrome has been directly associated with this pattern of pectoral nerve variation, brachial plexus abnormalities have been reported in congenital conditions involving neural crest cell dysfunction and limb development, such as neurocristopathies and Poland syndrome, suggesting that disruptions in early embryologic signaling and axonal guidance may contribute to variant branching patterns [8,10,19].

In our specimen, the unique innervation of both pectoral muscles resulted from a wide array of variants. Following convergence of variants, pectoral innervation came from a trunk-level pathway, bypassing the expected cord-level arrangement. Although the literature describes the lateral pectoral nerve (LPN) as a lateral cord branch (C5-C7) and the medial pectoral nerve (MPN) as a medial cord branch (C8-T1), cadaveric studies show significant variability in their anatomy. Loukas et al. found that the LPN originated from a single anterior division in up to 18% of specimens, as seen in our case [18,20]. Shetty et al., Prakash and Saniya, and Macchi et al. also described cases in which both pectoral nerves arose directly from the trunk divisions [11,17,21]. Harry et al. analyzed plexus variability at multiple levels, identifying variations in 16% of trunks, 4% of cords, and 25% of terminal branches - findings that are partially consistent with those observed in our specimen [15].

From an orthopedic perspective, this variation has possible clinical implications, since both LPN and MPN fibers originate from the anterior division of the middle trunk. Injury or surgical procedures at this level could cause complete pectoral denervation, especially during procedures such as shoulder arthroplasty, tendon repairs, clavicle fixation, proximal humerus fracture repair, and proximal clavicular tumor resections [15,22,23]. The deltopectoral approach used in arthroplasty and tendon repairs could risk damaging the LPN between the deltoid and pectoralis major. Damage to the LPN could weaken the pectoralis major, impairing adduction and internal rotation of the shoulder, thereby affecting postoperative rehabilitation and the patient's quality of life [24,25].

In our case, clavicle fixation carries considerable risks due to the potential presence of an aberrant trunk-level branch that may ascend medially into the surgical area, increasing the likelihood of transection during plate placement. This can lead to postoperative weakness in adduction or internal rotation, raising concerns about injury to the pectoral nerve [26,27]. While studies indicate the proximity of the brachial plexus to the clavicle, many practitioners focus on standard cord-level innervation, potentially underestimating the risks posed by proximal or trunk-level variants, as shown in this dissection. Additionally, fixing proximal humerus fractures, shoulder dislocations, and tendon transfers presents risks if atypical innervation is overlooked, especially in uncommon cases where pectoral muscle innervation may solely depend on an ansa pectoralis. Such an injury to a single proximal branch could result in complete functional denervation. Traditional studies often emphasize branching differences rather than converging into a single functional pathway, highlighting the need to appreciate the clinical significance of these variants [14,28].

This variation has attainable and important implications in other surgical fields, where preservation of pectoral nerve integrity is essential for maintaining chest wall function and optimizing reconstructive outcomes [29,30]. Compared with previous reports describing isolated variations in the origin or course of the pectoral nerve, our case illustrates a more integrated innervation pattern at the trunk level that may lead to greater functional deficits if injured [28]. Our variant innervation, which is superiorly located, could be affected during reconstructive shoulder surgery, compromising the viability of the pectoralis major myocutaneous flap and potentially influencing muscle tone, function, recovery, and the risk of adverse effects such as atrophy and myospasms during retraction or cauterization [25,31-38]. In anesthesiology, trunk-level origins, as in our case, might explain incomplete analgesia during brachial plexus or pectoral nerve (PECS I and II) blocks, as the branches may bypass standard anatomical landmarks [3]. In trauma surgery, injuries involving the anterior division of the middle trunk might cause more extensive pectoral paralysis than expected, possibly complicating the location and grafting of the lesion [39,40]. However, most available studies focus on typical branching patterns or singular irregularities. There remains a limited evaluation of how combined or convergent variants, such as those observed in our case, may affect surgical outcomes.

While this report does not support widespread changes in surgical practice, advancements in peripheral nerve imaging may create future opportunities for preoperatively identifying clinically significant brachial plexus variants. High-resolution imaging modalities may help clinicians evaluate peripheral nerve anatomy and guide surgical or anesthetic planning in select cases [41-44]. Recent literature suggests that modern 3T magnetic resonance neurography (MRN) suppresses surrounding signals to visualize peripheral nerves better, providing high-quality imaging of small neural structures, including complex brachial plexus anatomy and its variations, which may aid lesion localization and presurgical assessment [45]. Although surgical exploration remains the definitive standard for many complex plexus injuries, future advances in imaging resolution may improve the noninvasive identification of clinically relevant variants and support future reconstructive planning without substantially altering existing diagnostic workflows [45,46]. Among available modalities, high-resolution ultrasound may represent a particularly feasible and cost-effective approach for future investigation, given its accessibility, dynamic assessment capabilities, and increasing use in peripheral nerve evaluation. We understand that describing a single case may not support generalizability to the broader population. Therefore, documenting such cases will improve understanding of their prevalence and help develop strategies to prevent nerve injuries during surgical procedures [24,47].

## Conclusions

The case presented an atypical brachial plexus variation in which the lateral pectoral nerve arises from the anterior division of the middle trunk, forming an ansa pectoralis that gives rise to the medial pectoral nerve without the expected cord-level input. Although uncommon, this configuration may have implications in some surgical procedures, where inadvertent injury could alter pectoralis muscle function. Preoperative imaging is typically included in the standard of care; however, it may be helpful for healthcare providers to consult a clinical anatomist for an in-depth anatomical review of the affected area, which could aid in reducing surgical complications. Awareness of such a variant can reduce the risk of iatrogenic injury. There is a need for further research on how these anatomical variations may affect surgical outcomes, as most studies focus on typical branching patterns. Continued documentation of these anomalies will further enhance anatomical understanding and may aid in surgical procedure outcomes.
